# Characterization, Antimicrobial Activity, and Antioxidant Efficacy of a Pomegranate Peel Solution Against Persistent Root Canal Pathogens

**DOI:** 10.7759/cureus.43142

**Published:** 2023-08-08

**Authors:** Julia A Gallas, Laís L Pelozo, Wanderley P Oliveira, Sérgio L Salvador, Silmara M Corona, Aline E Souza-Gabriel

**Affiliations:** 1 Department of Restorative Dentistry, School of Dentistry of Ribeirão Preto, University of São Paulo, Ribeirão Preto, BRA; 2 Department of Pharmaceutical Sciences, School of Pharmaceutical Sciences of Ribeirão Preto, University of São Paulo, Ribeirão Preto, BRA; 3 Department of Toxicological and Bromatological Clinical Analyses, School of Pharmaceutical Sciences of Ribeirão Preto, University of São Paulo, Ribeirão Preto, BRA

**Keywords:** punica granatum, pomegranate extract, endodontic infections, antioxidant, antimicrobial

## Abstract

Background: The limitations of dental irrigation solutions reinforce the need to explore novel bioactive compounds that are safer and biodegradable. This study aimed to prepare a 10% pomegranate peel solution (*Punica granatum* extract - PGE) and evaluate its antimicrobial and antioxidant effects for root canal treatment.

Methods: Lyophilized extracts (1g/10 mL) from pomegranate peels were prepared, and the punicalagin content was assessed by ultra-performance liquid chromatography using pure punicalagin (standard). The antimicrobial activity was tested against common persistent root canal pathogens by the agar diffusion method, minimum inhibitory concentration (MIC), and minimum bactericidal/fungicide concentration (MCB/MFC). The antioxidant activity (%AA) was assessed by the DPPH radical scavenging method. Data were analyzed by ANOVA and Tukey's test (α = 0.05).

Results: The total phenolic content of the PGEextract was 6.55 µg/mL. Differences were found among the inhibition zone of PGE (23.32 ± 3.65), 1% NaOCl (30.76 ± 4.73), and 50% ethanol (without inhibition) (p < 0.05). The MIC values of PGE ranged between 6.25 and 75 mg/ml, and PGE was effective against the tested pathogens. PGE had antioxidant potential (IC50 = 3.52 µg/mL); however, the mean values were inferior to that of the quercetin (positive control) (IC50 = 0.95 µg/mL). The DPPH scavenging effect (%AA) of PGE (70.98 ± 2.3) had no difference from the positive control (72.94 ± 2.1) (p = 0.253).

Conclusion: The PGE extract was successfully biosynthesized and exhibited antimicrobial and antioxidant activity, suggesting its potential use as an adjuvant therapy during root canal treatment.

## Introduction

Polymicrobial infection and its products in the pulp tissue are responsible for periarticular damage and preoperative pain [1-3]. The persistence of pathogenic bacteria and the complexity of root canal anatomy directly influence the treatment outcomes [2,4,5].

Persistent infections are usually characterized by the presence of primarily resistant gram-positive facultative anaerobes, including *Streptococcus mutans*, *Staphylococcus aureus*, and *Enterococcus faecalis*, and some gram-negative strains such as *Escherichia coli* and *Candida albicans* fungus [2,3,5]. The success of root canal treatment depends on chemo-mechanical debridement, using solutions and endodontic files, and efficient tridimensional sealing of the canal system [4,6,7].

Sodium hypochlorite (NaOCl) and chlorhexidine gluconate (CHX) are the most common irrigation solutions used in root canal treatments [4,6]. NaOCl is widely used due to its high antibacterial activity and tissue dissolution ability [4,8], while CHX has broad-spectrum antimicrobial activity and substantivity [8,9].

However, NaOCl is highly cytotoxic and can produce allergic reactions [4,7,10]. It may also cause changes in the chemical composition of the dentin and collagen biodegradation and reduce the mechanical properties of dentin [6,8]. CHX lacks the tissue-dissolving property and has a high rate of dilution and degradation [4,11]. Besides, none of them has antioxidant activity, and both are incapable of removing the smear layer, demanding a chelating agent to improve the filling quality and sealer penetration [4,12].

Adjuvant therapies have been evaluated to improve the canal system disinfection and root filling quality [2,4-6]. Natural bioactive products have been tested for antioxidant and antimicrobial properties [7-9,13-17]. Applying an antioxidant agent before root canal filling can improve the sealer bond strength and stabilize the dentin collagen matrix [6].

Pomegranate fruits are widely consumed fresh and in commercial products, such as juices, jams, and wines [14,18]. The pericarp, seed liquid, and shell have polyphenols, polyphenolic acids, and tannins with astringent and anticancer properties [14,19], antioxidant activity [13,17], anti-inflammatory effect [19,20], and antimicrobial activity [17,21,22]. Besides, the extract improves dentin bond strength by reducing collagen degradation, inhibiting metalloproteinases, and removing the smear layer [23,24], suggesting a quality improvement of the root canal filling with resin-based sealers. Over half of the weight of the fruit belongs to the pomegranate peels, which are discharged as agro-industrial waste [17].

Previous studies have evaluated the antimicrobial and antioxidant activities of pomegranate peel extracts [4,8,13,16,17,25], including two studies that tested it inside the root canal system as an intracanal medicament [4,8]. Most studies have used an aqueous extract instead of hydroalcoholic [4,8,17], which can compromise the amount of phytochemicals in the solution. Some still need to perform the chemical characterization of the extract [4,8,25], which can directly influence their pharmacological activities [26]. Others did not evaluate the pomegranate solution against persistent root canal pathogens [16,17,25].

Considering the abovementioned facts, this study aimed to characterize a new pomegranate peel solution and evaluate the antimicrobial and antioxidant effect of the extract, intending to use it as adjuvant therapy in root canal treatment.

## Materials and methods

Preparation of the *Punica granatum* extract (PGE)

The study was approved by the Research Ethics Committee of the Ribeirão Preto School of Dentistry, University of São Paulo, Brazil (#61743716.0.0000.5419). Pomegranate ripe fruits were obtained from trees of a vegetative propagation grown in Ribeirão Preto, SP, Brazil. The extract was prepared after weighing and drying the peels by the freeze-drying method at -80 °C for 48h. The powder was stored at 4°C.

The powdered pomegranate peel (1g) was mixed with 10 mL of 50% ethanol in an ultrasonic bath for 40 min at 60 °C and 15.000 rpm. The extract was filtered and evaporated to dryness by a rotary evaporator (Büchi R-210, Flawil, Switzerland) [14]. The diluent used to prepare the extract was previously analyzed for antimicrobial activity at several hydroalcoholic concentrations, and 50% ethanol did not inhibit microbial growth.

Identification and chemical characterization using ultra-performance liquid chromatography (UPLC)

Determination of the punicalagin content in the PGE was carried out by UPLC using pure punicalagin as the standard (Sigma-Aldrich, St Louis, USA) [16].

The UPLC data were acquired on a Shimadzu UPLC system (Kyoto, Japan) at 280 nm with a diode array detector (Shimadzu, SPD-M20-A) via an Ascentis Express column (2.7 µm; 4.6 × 100 mm; Waters Inc., Milford, USA) and a Xevo TQ-S micro mass spectrometer (Waters Corporation, Milford, USA), operating with a Z-spray ionization source.

The following parameters were used for the detection: selected ion recording mode; capillary voltage = 2.50 kV; cone voltage = 40 V; source temperature = 150 °C; desolvation temperature = 400 °C; and desolvation gas = 300 L/h. The mobile phase used was 0.1% formic acid (A) and 0.1% formic acid in acetonitrile (B), with a gradient of 10% methanol over 12 min at a flow rate of 500 μL/min.

Antimicrobial activity

The antimicrobial activity of the PGE was tested against gram-positive strains (*Enterococcus faecalis*, *Staphylococcus aureus,* and *Streptococcus mutans*), gram-negative strains (*Escherichia coli*), and fungal strains (*Candida albicans*) by the agar well diffusion method. Before plating on culture plates, a 24-hour culture was used to prepare the microbial suspension (BHI) for the five strains. It was adjusted to an optical density of 0.5 McFarland standard for bacteria and 2 for the fungal strain. The microbial suspensions were evenly spread on the surface of 90 x 15 mm petri plates using a sterile swab, and the agar plates were incubated for 24 hours at 37 °C. The bacterial growth curve was determined on two replicate plates per strain, and the absorbance was recorded with a spectrophotometer (Femto 432; Marconi, Piracicaba, Brazil) at 600 nm.

The inhibition zone was determined for the diffusion test in agar in 90 x 15 mm petri plates and 1 ml of the microbial inoculum was added to the plates. A sterile test tube was used to bore three 15 mm diameter holes in the agar plates, and the holes were filled with 250 μL of the PGE 1% NaOCl (positive control), and 50% ethanol solution (negative control). The agar plates were incubated for 24 hours at 37 °C, and the tests with the solutions were performed in duplicate, on different days [10].

The PGE was transferred to Eppendorf tubes and sequentially diluted in deionized water by 10-fold serial dilution to determine the minimum inhibitory concentration (MIC). Then, 90 mL of a standardized culture of each bacterial strain was mixed with 10 μl of the microbial inoculum and 100 μL of each dilution of the extracts and dispensed on 96 microplate wells. After incubation at 37 °C for 24 hours, 50 μL of a viability reagent (0.125% alamarBlue) was added to each well to assess cell viability. The minimal inhibitory concentration (MIC) was determined on three replicate microplates for each microorganism, and it was described as the lowest concentration of the extract that inhibited the microorganisms' visible growth [21,23].

For the minimum bactericidal/fungicide concentration (MCB/MFC) tests, 20 μL of each microplate well solution with dilutions of the PGE and microorganisms were spread on agar plates. Plates were evaluated on three replicate plates for microorganism growth after 24 h of incubation at 37 °C, and the concentration with no bacterial growth was determined as the MBC/MFC [10].

Antioxidant activity by DPPH radical scavenging

The DPPH (2,2-diphenyl-1-picrylhydrazyl) radical scavenging activity was measured using the Blois method [27]. Then, 10 μL of the PGE were prepared from previously described concentrations and mixed with a solution containing 1.0 mL of 0.1 M acetate buffer (pH 5.5), 1 mL ethanol, and 0.5 mL of DPPH∙ 0.250 mM ethanolic solution. Quercetin was used as the standard antioxidant solution (control).

The reaction mixture was incubated in the dark for 30 min at 25°C, and absorbance was recorded with a spectrophotometer (Shimadzu, Kyoto, Japan) at 517 nm. The DPPH scavenging effect (%AA) was calculated using the formula: $\frac{A (control)-A (sample)}{A (control)} \cdot 100$, where A (control) represents the absorbance of the DPPH solution without the PGE and A (sample) represents the absorbance of the extract with DPPH. The results were expressed as IC50 (µg/mL), which indicates the concentration of solution required to produce a 50% reduction in the DPPH free radical, in triplicate measurements.

Data analysis

Statistical analysis was done with IBM SPSS Statistics for Windows, Version 25 (Released 2017; IBM Corp., Armonk, New York, United States), with a 5% significance for all tests. Shapiro-Wilk and Levene's tests were used to check the normality and homogeneity of data. One-way ANOVA and Tukey's tests analyzed the agar diffusion test and the antioxidant activity. The MIC and MBC/MFC were qualitatively analyzed based on microbial growth.

## Results

UPLC analysis

**Figure 1:**
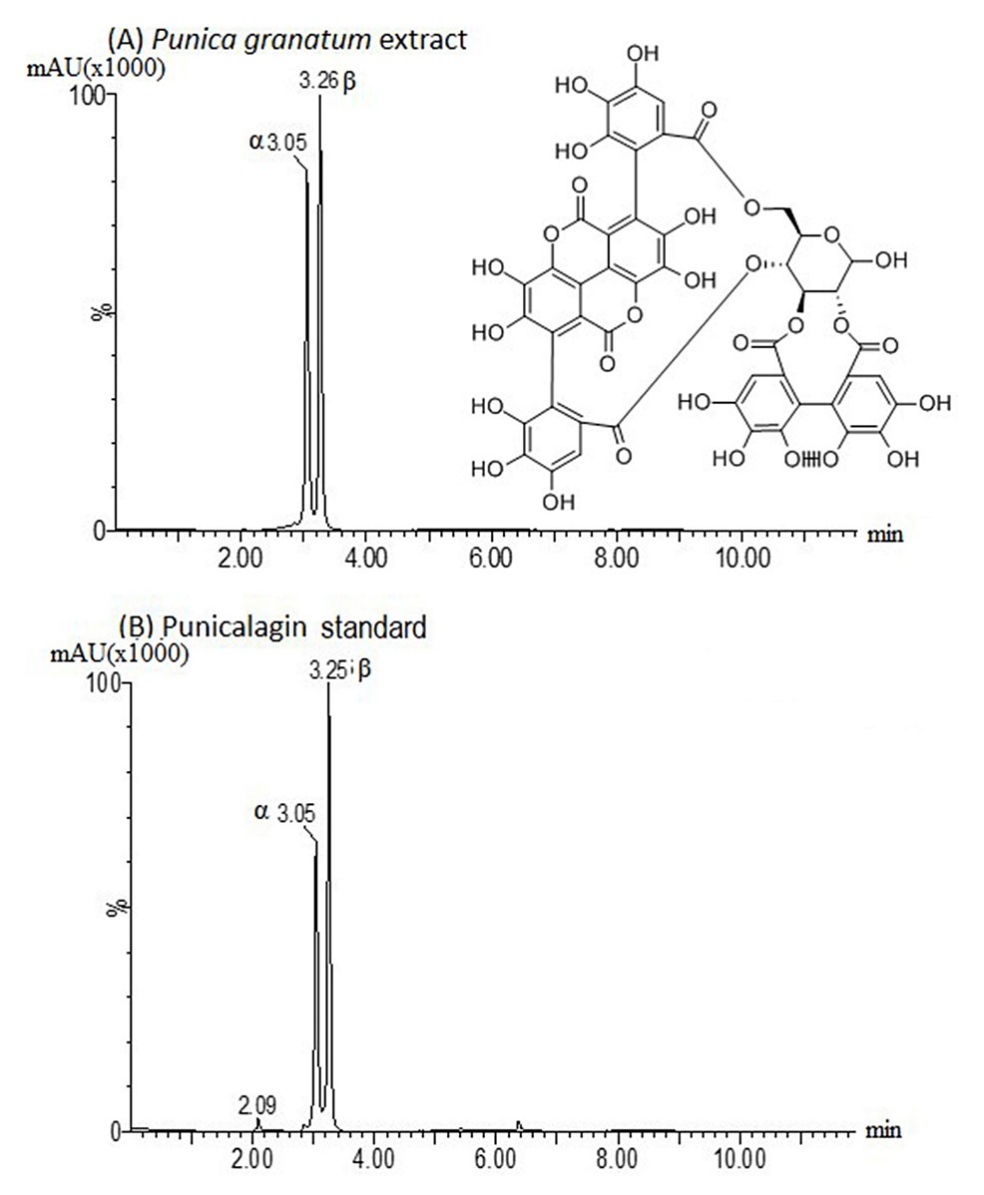
Chromatograms of the tested solutions. Note the similar punicalagin peaks of the ellagitannin isomers on a hydroalcoholic extract of the pomegranate peel (A) and punicalagin standard (Sigma-Aldrich) (B).

The UPLC chromatogram showed two significant punicalagin peaks for ellagitannin isomers of the experimental PGE (α = 3.05 and β =3.25) with no difference for the punicalagin standard (control: α = 3.05 and β =3.26). The total phenolic content of the experimental PGE was found as 6.55 µg/mL, with 3.05 µg/mL for the α isomer and 3.5 µg/mL for the β isomer (Figure 1).

Antimicrobial analysis

The diffusion agar test showed differences between the zone of inhibition means obtained for the PGE (23.32 ± 3.65b), 1% NaOCl (positive control) (30.76 ± 4.73a), and the 50% ethanol solution (negative control) that had no zone of inhibition (p < 0.05) (Table 1).

**Table 1:** Zone of inhibition means (mm) and standard deviations (SD) obtained for pomegranate extract (experimental) and sodium hypochlorite (positive control). *ATCC: American Type Culture Collection. Different letters indicate a significant difference (α = 0.05) within each line (comparison of the solutions' effect for the same strain)

Microorganism	ATCC*	*Punica granatum* extract	1% sodium hypochlorite
Enterococcus faecalis	4683	(21.56 ± 2.89) b	(29.01 ± 1.67) a
Staphylococcus aureus	6538	(25.23 ± 4.07) b	(32.89 ± 1.62) a
Streptococcus mutans	25175	(20.13 ± 1.91) a	(24.93 ± 1.99) a
Escherichia coli	8739	(24.53 ± 3.37) b	(29.13 ± 2.04) a
Candida albicans	1023	(25.19 ± 2.97) b	(37.81 ± 1.90) a
Total		(23.32 ± 3.65) b	(30.76 ± 4.73) a

**Figure 2:**
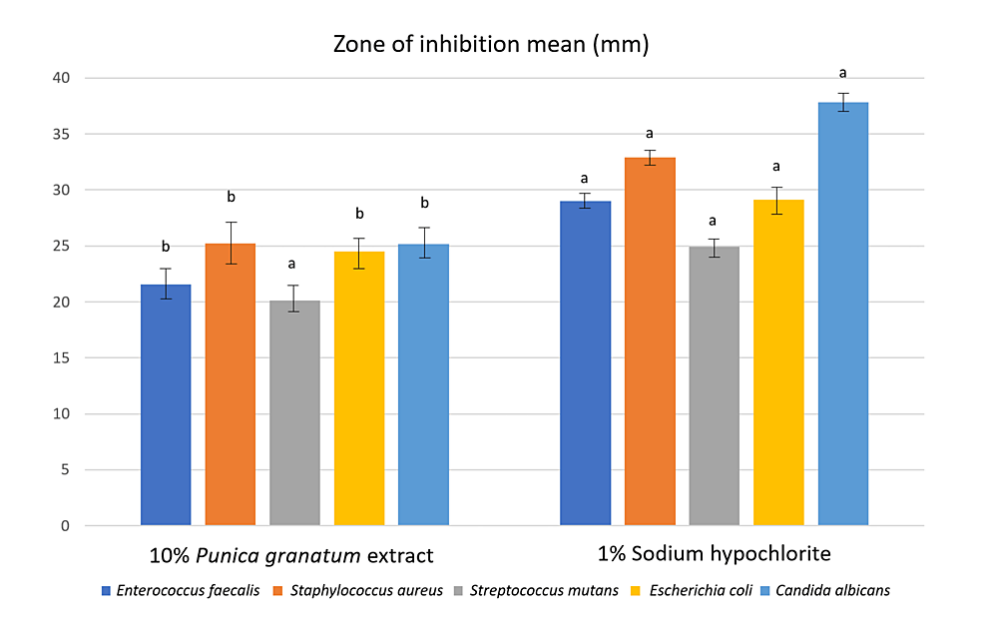
Graphical representation of the inhibition zones caused by the pomegranate extract and 1% sodium hypochlorite against persistent root canal pathogens. Different letters indicate a significant difference between the solutions for the same strain (α = 0.05).

Regardless of the microorganism, larger zones of inhibition diameter were observed for the 1% NaOCl when compared with the PGE (p < 0.05). However, for the *Streptococcus mutans*, the PGE had no difference from NaOCl solution (p = 0.000) (Figure 2).

The MIC values of PGE ranged between 6.25 and 75 µg/ml against all the persistent root canal pathogens (Table 2).

**Table 2:** Minimum inhibitory concentration and minimum bactericidal/fungicide concentration for the pomegranate extract against persistent root canal pathogens (μg/mL). *MIC: Minimum inhibitory concentration; ^#^MBC/MFC: minimum bactericidal concentration/minimum fungicide concentration

Microorganism	MIC*	MCB/MFC^#^
Enterococcus faecalis	75	75
Staphylococcus aureus	12.5	12.5
Streptococcus mutans	50	75
Escherichia coli	25	50
Candida albicans	6.25	12.5

The experimental extract was recorded with the lowest MIC value of 6.25 µg/ml for *Candida albicans* strain.

Antioxidant activity

**Figure 3:**
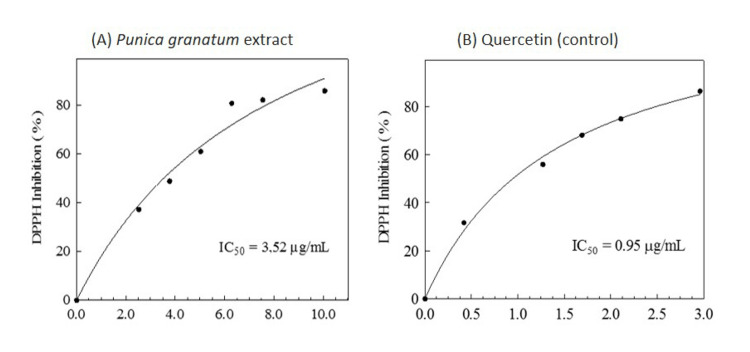
Antioxidant curves of the experimental and control solutions using the DPPH radical capture method: (A) pomegranate peel extract (IC50 = 3.52 µg/mL); (B) quercetin (control, IC50 = 0.95 µg/mL) The IC50values indicate the concentration of solution required to produce a 50% reduction in the DPPH free radical

The results of the DPPH radical scavenging activity showed that the PGE had antioxidant potential (IC50 of PGE = 3.52 𝜇g/mL); however, it was inferior when compared with quercetin (positive control) (IC50 of control = 0.95 𝜇g/mL) (Figure 3).

The DPPH scavenging effect (%AA) or percent inhibition of PGE (70.98 ± 2.3) was statistically similar to those obtained for quercetin (72.94 ± 2.1) (p = 0.253).

## Discussion

The limitations of the standard irrigation solutions reinforce an urgent need to explore natural active compounds that are safer and biocompatible. These novel solutions could also supply other desired properties, such as non-toxicity, antioxidant activity, and the ability to remove debris from dental surfaces [7,8].

The polyphenolic and tannin compounds (as the punicalagin) promote the antimicrobial activity of the PGE through the precipitation of proteins on the cell surface of the microorganism and inhibiting metabolic enzymes that cause cell lysis [16,28]. The preparation method and chemical characterization of tested extracts are essential because their activity is directly affected by the plant part, plant age, season of collection, drying strategy, and extraction procedure [3].

The UPLC test showed the presence of α-punicalagin and β-punicalagin with two almost similar peaks in the UV spectrum compared to the positive control. This result corroborates with the findings by Celiksoy et al., who found two similar punicalagin peaks in the UPLC chromatogram [16]. However, the total amount of phenolic content was different between researchers. While our study found 6.55 µg/mL, Celiksoy et al. reported 170 mg/g of punicalagin in their pomegranate extract [16].

Previous studies found antimicrobial properties of ethanolic and methanolic extracts of the PGE against oral microorganisms [10,29]. In our study, 50% ethanol was used during extraction because ethanomeric mixtures were more appropriate for human consumption and had no antimicrobial activity at this concentration [10,30]. The lack of antimicrobial activity allowed us to elucidate the PGE activity without interference. The peel was chosen for the extract due to its high polyphenols and flavonoid contents [24].

The diffusion agar test showed less bacterial inhibition ability of the PGE when compared with 1% NaOCl. However, it had a positive inhibition effect on persistent root canal pathogens' growth, and it was even similar to the NaOCl for the *Streptococcus mutans*. These findings are in accordance with those of Sisodiya et al. [8], who also found the best inhibition zones for NaOCl against *Enterococcus faecalis* and with Hanafy et al. [25] who found inhibition zones between 20 and 25 mm for the PGE ethanolic extract against all tested strains. Conversely, Mallya et al. found the largest inhibition zone with the combination of 20% aqueous extract of PGE and CHX [4].

The inferior bacterial inhibition of the PGE was an expected outcome since the comparison was made with the most effective ‘gold standard’ antimicrobial solution (NaOCl). Further studies should compare the natural extract with water or CHX. CHX can be used as an alternative to NaOCl, especially in cases of open apex, root resorption, foramen enlargement, and root perforation, due to its biocompatibility, the high cytotoxicity of NaOCl, or in cases of allergy related to bleaching solutions [4,7,10]. For this reason, a future study combining the PGE and CHX might be interesting.

The MIC and MCB/MFC values show the antibacterial and antifungal abilities of the PGE. Variations of MIC values remaining below 75 µg/ml against the oral pathogens suggest the effective activity of the experimental extract to be used as an adjuvant in root canal treatment. The MIC values of 6.25 and 75 µg/mL against *Candida albicans* and *Enterococcus faecalis* corroborate with the findings of Mallya et al. [4], who found the MIC to be 50 μg/mL of the peel extract against *Enterococcus faecalis *and with those of Sousa et al. [3] who found an MIC value of 15.62 µg/mL for both strains with the PGE leaf extract. These results also suggested a positive antimicrobial effect since natural products are considered potent inhibitors of microbial activity when MIC values are lower than 500 μg/mL.

The PGE showed a more effective antimicrobial effect against the *Streptococcus​​​​​​​ mutans* that was similar to NaOCl, according to Hanafy et al. [25], who observed a higher inhibitory effect against all tested gram-positive food-borne pathogens. Other authors [22] found that the PGE killed *Streptococcus*​​​​​​​* mutans* at high concentrations and reduced biofilm and acid production, so it could potentially prevent dental caries [15,18].

The inhibitory effect of the PGE can be explained by the polyphenolic ability to increase the bacterial membrane's breakdown [4,21], inhibiting their co-aggregation and growth. Moreover, natural polyphenols can stimulate the deprivation of substrate in microbial metabolism through oxidative phosphorylase [19,22]. The action mechanism of tannins on yeasts is still unknown, but it can be related to cell walls and cytoplasmic membrane injury [16,20].

Punicalagin is responsible for almost 50% of the PGE antioxidative potential [14,19]. The %AA was evaluated using the DPPH free radical method, based on how the antioxidants bind with DPPH, a stable organic radical [27]. Our study found antioxidant activity for PGE (IC50 = 3.52 µg/mL) similar to the positive control (quercetin). This result corroborates with earlier studies [13,17,25], and it may be considered a good antioxidant potential (IC50 < 4.0 𝜇g/mL) [27].

The antioxidant potential of the PGE can be attributed to the phenolic compounds, which can scavenge free radicals, act as hydrogen donors, and inhibit the oxidative process [19,27,28]. The inhibitory effect of the PGE against MMPs suggests a preventive potential against collagen loss [28]. Besides, the ethanolic PGE is non-toxic to human tissues, which can be advantageous over standard irrigation solutions [14].

Another advantage is that plant parts have capping, stabilizing, reducing, and oxidative agents, promoting their synthesis [17]. Antioxidants can also modify the dental surface, improving the polymerization of adhesive and resin-based materials and directly influencing the hybrid layer's stability by creating a suitable substrate for the restorative procedure [24]. It suggests a possible positive effect of the solution on the bond strength of resin-based sealers that should be elucidated.

The limitation of this study was that it only quantified the punicalagin content of the PGE. Suitable extraction and quantification procedures should be developed to recover and identify/quantify as many antioxidants as possible. The PGE had an antioxidant effect and inhibited the growth of persistent root canal pathogens; therefore, it can be used during root canal treatment. Furthermore, it can be a good alternative for dentin treatment before dental restorations. This laboratorial research opens possibilities to develop new formulations containing the PGE for further research to clarify the effects of this promising natural extract on restorative dentistry.

## Conclusions

Standard irrigation solutions have limitations, and the development of natural compounds can benefit root canal therapy. The experimental pomegranate peel extract had antimicrobial activity against the persistent root canal pathogens, although in an inferior amount than NaOCl. This was an expected outcome since the comparison was made with the most effective antimicrobial solution. For the *Streptococcus mutans*, both solutions had similar antimicrobial effects.

Besides the antimicrobial action, the pomegranate extract showed good antioxidant activity; therefore, it can be used as an alternative irrigation solution and has the potential to be included in commercially available products targeting oral therapy.
